# Intima‐Media Thickness Progression Rate and Primary Unassisted Patency Failure in Arteriovenous Fistulas: A Prospective Cohort Study

**DOI:** 10.1111/hdi.70084

**Published:** 2026-05-24

**Authors:** Gen Li, Zhengjiang Cao

**Affiliations:** ^1^ Department of Nephrology West China TianFu Hospital of Sichuan University Chengdu Sichuan China

## Abstract

**Background:**

Failure of autogenous arteriovenous fistulas (AV fistulas) due to venous neointimal hyperplasia is a major clinical challenge. Whether the rate of progression in venous intima‐media thickness predicts primary unassisted patency failure remains unknown.

**Methods:**

In this prospective cohort study, we performed a 6‐month landmark analysis to minimize immortal time bias among patients with newly created AV fistulas. Intima‐media thickness was measured serially by ultrasound. We calculated the early progression rate (0–6 months) as the primary predictor and the 0–12 month rate as a sensitivity analysis. Cox proportional hazards models were used to assess the association between the early progression rate and subsequent primary unassisted patency failure.

**Results:**

The analysis included 129 patients who were event‐free at the 6‐month landmark. Over a median follow‐up of 13.2 months after the landmark, 35 patients (27.1%) experienced primary unassisted patency failure. Faster early intima‐media thickness progression was a strong independent predictor of failure (adjusted hazard ratio [HR] 1.63 per 0.05 mm/year increase; 95% CI 1.41–1.88; *p* < 0.001), together with baseline intima‐media thickness and diabetes. Adding the early progression rate to a model containing clinical and baseline ultrasound variables significantly improved discrimination (C‐index increased from 0.69 to 0.76, *p* = 0.002) and risk reclassification (net reclassification improvement = 0.21, *p* = 0.008).

**Conclusions:**

Early venous intima‐media thickness progression rate is a powerful independent predictor of subsequent AV fistula failure and adds substantial prognostic value beyond standard clinical and ultrasound factors. Serial ultrasound monitoring of this parameter may support the development of risk‐adapted surveillance strategies to prolong AV fistula survival.

## 1 Introduction

The underlying pathophysiology of dysfunction in autogenous arteriovenous fistulas (AV fistulas) is predominantly characterized by maladaptive remodeling of the venous outflow tract. The abrupt hemodynamic changes induced by AV fistula creation, specifically, elevated blood flow and altered shear stress, trigger a cascade of cellular and molecular events, including endothelial activation, leukocyte recruitment, and proliferation of vascular smooth muscle cells. This process frequently culminates in neointimal hyperplasia and progressive venous stenosis [1, 2, 3]. Despite advancements in surgical techniques and preoperative mapping, this maladaptive response remains the primary driver of maturation failure and subsequent loss of primary unassisted patency, contributing to the substantial rate of dysfunction observed within the first year of access creation [4, 5, 6]. Systemic host factors prevalent in the dialysis population, such as diabetes mellitus, chronic inflammation, uremic toxins, and disordered mineral metabolism, further modulate this remodeling, contributing to the significant interindividual variability in AV fistula outcomes [1, 2, 3]. These mechanistic insights highlight the potential value of noninvasive imaging parameters that can quantitatively reflect the burden and longitudinal evolution of venous wall remodeling.

High‐resolution ultrasound measurement of intima‐media thickness is a well‐established and reproducible noninvasive metric for assessing arterial wall remodeling and cardiovascular risk, particularly in carotid and peripheral arteries [7]. Extending this imaging paradigm to the venous outflow segment of a matured AV fistula is biologically rational: venous intima‐media thickness can serve as an integrative marker of the cumulative impact of local hemodynamic stressors, cellular proliferation, and extracellular matrix changes. Crucially, while a single baseline intima‐media thickness measurement captures the structural state at one point in time, the longitudinal rate of change offers a personalized index of ongoing pathobiology. This progression metric potentially provides a more accurate and timelier forecast of impending stenosis or thrombosis compared to single‐time‐point measurements, aligning with the progressive nature of AV fistula remodeling.

Despite the strong biological rationale for venous intima‐media thickness as a marker of AV fistula remodeling, the clinical utility of its progression rate for predicting outcomes remains largely unestablished. Prior studies have often relied on single‐time‐point measurements, failing to capture the longitudinal evolution of remodeling [8, 9]. Even among the limited longitudinal investigations, methodological inconsistencies in ultrasound protocols, anatomical sampling sites, and analytical approaches for defining progression rates have hindered the robust characterization and generalizability of findings [9]. More critically, there is a distinct lack of comprehensive evidence demonstrating how this rate of progression, beyond basic statistical association, can significantly improve patient‐specific risk stratification. Consequently, despite its mechanistic appeal, the potential of serial intima‐media thickness assessment as a practical parameter for risk‐adapted surveillance in AV fistula management has not been fully realized [10].

To address these critical knowledge gaps, we conducted a prospective cohort study of patients undergoing native AV fistula creation. Our primary objectives were to: (1) characterize the temporal trajectory of intima‐media thickness in the venous outflow tract after AV fistula maturation using standardized, serial high‐resolution ultrasound measurements; (2) prospectively quantify individual intima‐media thickness progression rates; and (3) rigorously evaluate whether this progression rate independently predicts primary unassisted patency failure and significantly improves overall prognostic performance when integrated with conventional clinical predictors. We hypothesized that a faster progression rate would be independently associated with a higher risk of primary access failure and that its incorporation into risk models would yield clinically meaningful improvements in patient stratification.

## 2 Materials and Methods

### 2.1 Study Design and Participants

This prospective, single‐center, observational cohort study was conducted at West China TianFu Hospital of Sichuan University and approved by the Institutional Review Board. All participants provided written informed consent.

Consecutive adult patients (≥ 18 years) scheduled for new native AV fistula creation for maintenance hemodialysis between January 2024 and June 2024 were enrolled. Eligible AV fistula types included radiocephalic, brachiocephalic, and brachiobasilic fistulas. Exclusion criteria were a prior failed AV fistula in the same limb, known central venous obstruction, a life expectancy of less than 3 months, or inability to provide consent.

This study employed a landmark analysis to evaluate the prognostic value of the early intima‐media thickness progression rate while mitigating immortal time bias. The primary landmark time point was set at 6 months after the first successful cannulation of the mature AV fistula. The main analysis cohort comprised patients from the initial cohort who were alive and free of a primary unassisted patency failure event at this 6‐month landmark.

### 2.2 Clinical Data Collection and Follow‐Up

All AV fistulas were created by experienced vascular surgeons following standard institutional protocols. An AV fistula was considered mature upon successful cannulation for hemodialysis on at least six occasions. The date of the first successful cannulation marked the “baseline” (time zero) for post‐maturation follow‐up.

Baseline demographic data, comorbidities (including diabetes mellitus, hypertension, and coronary artery disease), dialysis vintage, and pre‐operative laboratory parameters were collected from electronic medical records. Patients underwent routine clinical surveillance of their AV fistula, including physical examination at each dialysis session and duplex ultrasound as per center protocol.

### 2.3 Ultrasound Protocol and Intima‐Media Thickness Measurement

Serial high‐resolution B‐mode ultrasound examinations were performed by certified sonographers blinded to clinical outcomes using a Philips Epiq 7G ultrasound system (Philips Healthcare, Andover, MA, USA) with a high‐frequency L12‐5 linear array transducer (5–12 MHz). Scans were scheduled at baseline (maturation), 3 months, and 6 months, with subsequent annual scans.

Intima‐media thickness was measured in the venous outflow tract at a standardized, reproducible site. For radiocephalic AV fistulas, the measurement was taken within the outflow vein, 2 cm distal to the anastomosis (in the direction of blood flow). For brachiocephalic and brachiobasilic types, measurements were taken at a fixed point 2 cm proximal to the antecubital fossa. This juxta‐anastomotic site was chosen to ensure reproducibility while avoiding extreme hemodynamic turbulence immediately adjacent to the surgical connection. At each visit, three measurements of the far wall thickness were obtained from a longitudinal view within a 10‐mm segment free of plaque. The thickness was defined as the distance from the lumen‐intima interface to the media‐adventitia interface, with the average of triplet measurements recorded as the final value for that visit.

### 2.4 Calculation of Progression Rates

For each patient, the “early intima‐media thickness progression rate” was calculated using measurements from baseline, 3 months, and 6 months. This rate was defined as the slope of the linear regression of thickness versus time (in years). Similarly, the “late progression rate” was defined as the longitudinal slope spanning from baseline to 12 months for sensitivity analyses. The rates are presented in mm/year and were modeled as continuous variables (per 0.05 mm/year increment, representing approximately half of the interquartile range) in survival analyses.

### 2.5 Outcome Definition

The primary outcome was primary unassisted patency failure, defined as the first occurrence of: (1) any endovascular or surgical intervention performed to maintain or restore patency (including angioplasty for hemodynamically significant stenosis), or (2) thrombotic occlusion resulting in access abandonment.

For the landmark analysis, follow‐up for the outcome began at the 6‐month landmark date. Once a patient reached an endpoint (intervention or occlusion), they were considered to have failed primary unassisted patency, and no further ultrasound measurements were included in the progression analysis to avoid bias. Patients were censored at the time of death, kidney transplantation, transfer to a different dialysis facility, or the end of the study follow‐up period. The progression rate was calculated post hoc and blinded to the clinical team. Interventions were performed strictly according to KDOQI guidelines based on clinical signs and hemodynamics, independent of the intima‐media thickness progression rate.

### 2.6 Statistical Analysis

Statistical analyses were conducted using SPSS version 26.0 (IBM Corp.) and R version 4.2.2. Continuous variables are presented as mean ± standard deviation or median, and categorical variables as counts (percentages).

Cox proportional hazards regression was used to assess the association between predictors and time to primary unassisted patency failure from the 6‐month landmark. Residual plots and Schoenfeld residuals were used to confirm the proportional hazards assumption. A multivariable model was built to adjust for clinically relevant confounders, regardless of their statistical significance in univariable analysis, including age, diabetes, dialysis vintage, initial venous diameter, and baseline intima‐media thickness.

The incremental prognostic value of the intima‐media thickness progression rate was evaluated by comparing a baseline model (clinical factors and baseline intima‐media thickness) with an extended model (baseline model plus progression rate). We assessed improvements in discrimination using Harrell's C‐index and the area under the time‐dependent receiver operating characteristic curve at 1 year. Risk reclassification was quantified using the continuous net reclassification improvement and the integrated discrimination improvement. The clinical utility and net benefit were evaluated using decision curve analysis. A secondary landmark analysis at 12 months was performed as a sensitivity analysis. All statistical tests were two‐sided, and a *p*‐value < 0.05 was considered statistically significant.

## 3 Results

### 3.1 Study Population and Landmark Cohort

The participant selection and study flow are detailed in Figure 1. Of the 158 patients initially assessed for eligibility, 150 met the inclusion criteria and formed the initial study cohort. Following the 6‐month calculation period for the early intima‐media thickness progression rate, 129 patients (86.0%) were event‐free and were included in the primary landmark analysis.

**FIGURE 1:**
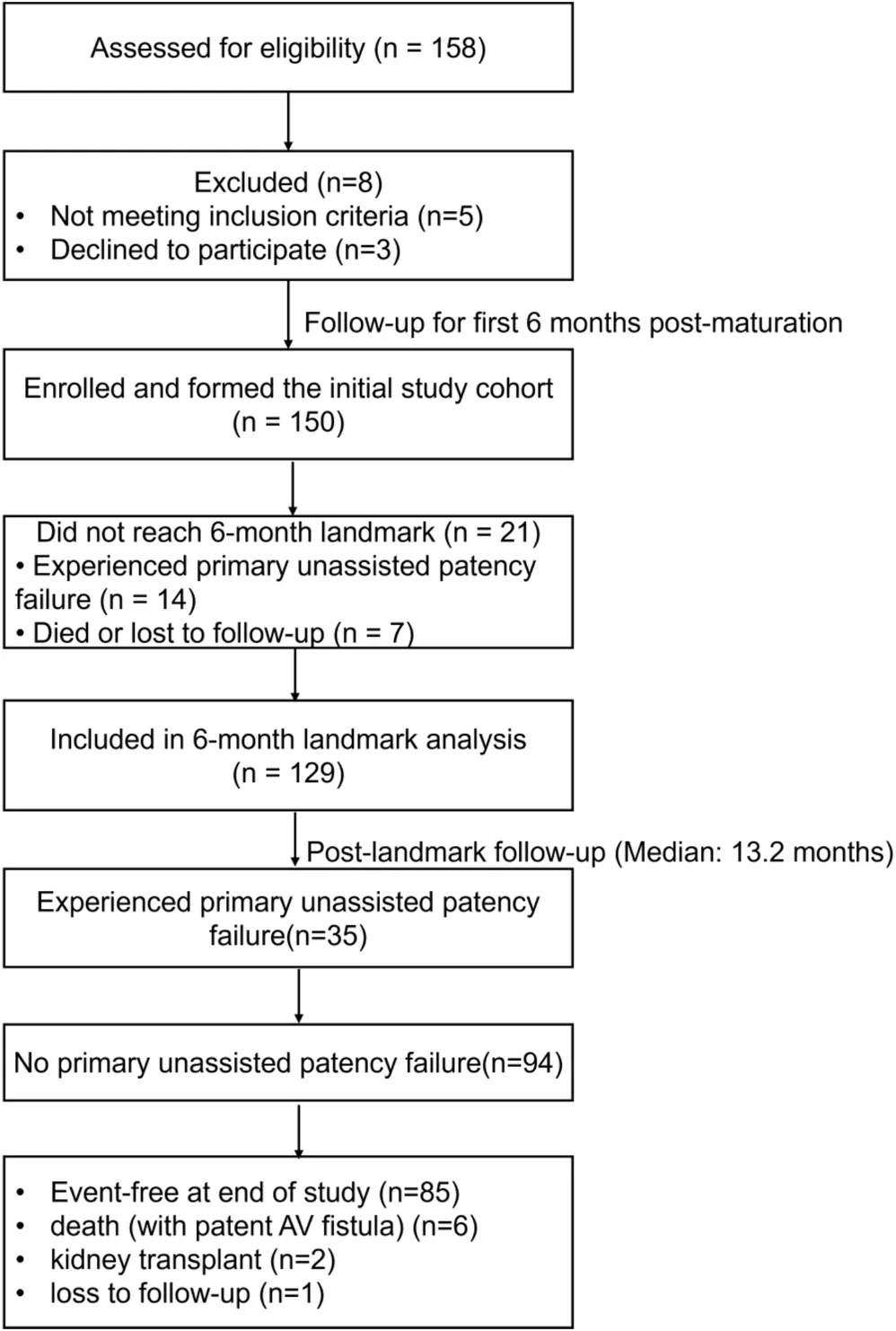
Flow diagram of study participants. The flowchart details the patient selection process, follow‐up, and key study procedures. Of the 158 patients assessed for eligibility, 150 were enrolled and underwent serial ultrasound measurements to determine the intima‐media thickness progression rate. A total of 129 patients were event‐free at the 6‐month landmark and were included in the final analysis for predicting primary unassisted patency failure.

During a median post‐landmark follow‐up of 13.2 months (interquartile range 10.5–15.8 months), 35 of the 129 patients (27.1%) experienced primary unassisted patency failure. The baseline characteristics of this landmark cohort, stratified by their subsequent failure status, are presented in Table 1. Patients who eventually developed patency failure had a significantly higher prevalence of diabetes mellitus (62.9% vs. 39.4%; *p* = 0.021), a greater baseline venous thickness (0.57 ± 0.12 mm vs. 0.51 ± 0.10 mm; *p* = 0.008), and a markedly faster early progression rate (median 0.19 vs. 0.12 mm/year; *p* < 0.001) compared to those who did not.

**TABLE 1:** Baseline characteristics of the 6‐month landmark cohort (*n* = 129), stratified by post‐landmark primary unassisted patency failure.

Characteristic	Total cohort (*n* = 129)	No failure event (*n* = 94)	Patency failure event (*n* = 35)	*p*
Demographics				
Age, years	62.8 ± 10.5	62.5 ± 10.8	63.5 ± 9.8	0.65
Male sex	74 (57.4)	53 (56.4)	21 (60.0)	0.73
Body mass index, kg/m^2^	23.9 ± 3.1	24.0 ± 3.2	23.7 ± 2.9	0.68
Dialysis vintage, years	3.4 [1.8–5.8]	3.3 [1.8–5.5]	3.6 [2.0–6.2]	0.55
Comorbidities				
Hypertension	110 (85.3)	80 (85.1)	30 (85.7)	0.93
Diabetes mellitus	59 (45.7)	37 (39.4)	22 (62.9)	0.021
Coronary artery disease	31 (24.0)	22 (23.4)	9 (25.7)	0.81
Laboratory parameters (at baseline)				
Hemoglobin, g/L	98.5 ± 12.0	99.0 ± 11.5	97.1 ± 13.2	0.48
Serum albumin, g/L	36.7 ± 3.8	37.0 ± 3.5	35.9 ± 4.4	0.21
hs‐CRP, mg/L	6.8 [3.3–11.5]	6.5 [3.0–11.0]	7.5 [4.0–12.8]	0.25
AV fistula characteristics				
AV fistula type (RC)	91 (70.5)	66 (70.2)	25 (71.4)	0.90
Initial venous diameter, mm	2.6 ± 0.5	2.7 ± 0.5	2.5 ± 0.4	0.11
Key ultrasound predictors				
Baseline Intima‐Media Thickness, mm	0.53 ± 0.11	0.51 ± 0.10	0.57 ± 0.12	0.008
Early intima‐media thickness progression rate (0–6 months), mm/year^a^	0.14 [0.10–0.20]	0.12 [0.08–0.15]	0.19 [0.16–0.25]	< 0.001

^a^ Early intima‐media thickness progression rate was calculated for each patient using ultrasound data from baseline, 3 months, and 6 months.

### 3.2 Longitudinal Changes and Distribution of Early Progression

In the full cohort of 150 patients, the mean venous intima‐media thickness increased steadily from 0.53 ± 0.11 mm at maturation (baseline) to 0.57 ± 0.12 mm at 3 months and 0.60 ± 0.13 mm at 6 months. The distributions of these serial measurements are illustrated in Figure 2. The reproducibility of measurement was excellent, with an intra‐observer intraclass correlation coefficient of 0.92 (95% CI 0.88–0.95) and an inter‐observer coefficient of 0.90 (95% CI 0.86–0.94).

**FIGURE 2:**
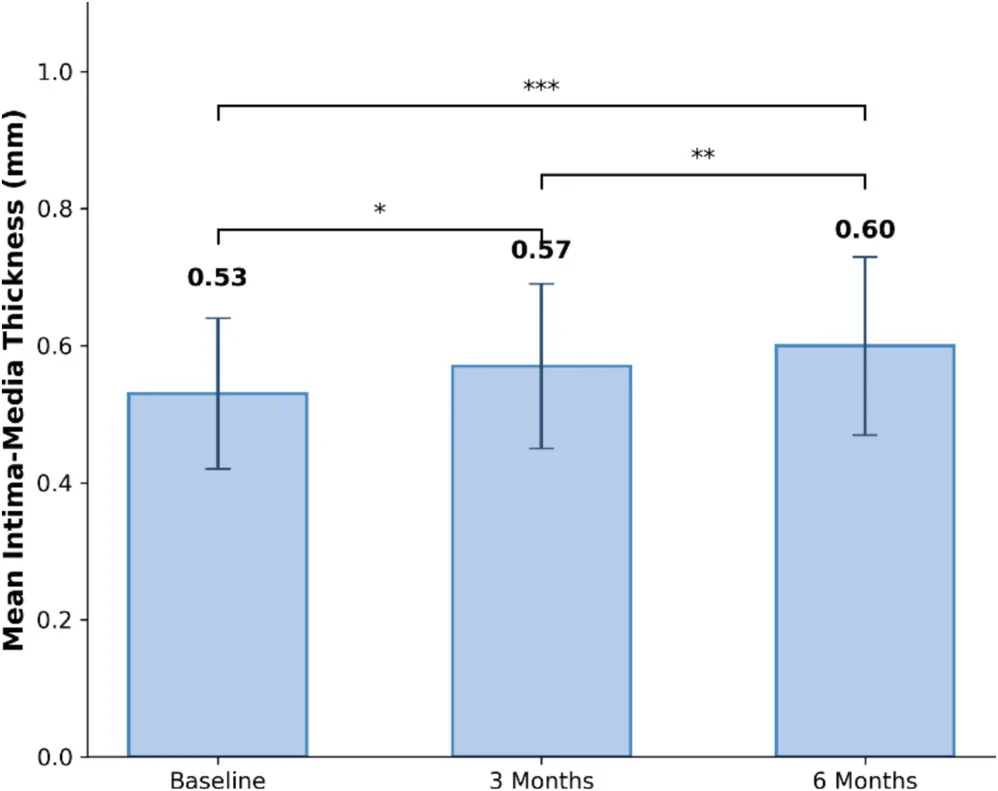
Progressive and significant increase of venous intima‐media thickness over the first 6 months. The box plot displays the distribution of venous intima‐media thickness at baseline, 3 months, and 6 months. The central line represents the median, the box represents the interquartile range, and the whiskers extend to 1.5 times the interquartile range. *p < 0.05, **p < 0.01, ***p < 0.001.

Within the 6‐month landmark cohort (*n* = 129), the early progression rate, calculated using data from 0 to 6 months, showed a right‐skewed distribution with a median of 0.14 mm/year (interquartile range 0.10–0.20 mm/year). For analytical purposes, patients were categorized into tertiles based on this early rate: Low (< 0.10 mm/year; *n* = 43), Moderate (0.10–0.18 mm/year; *n* = 43), and High (> 0.18 mm/year; *n* = 43).

### 3.3 Patency Outcomes in the Landmark Cohort

At the 6‐month landmark time point, 129 of the initial 150 patients had a patent AV fistula and remained in the study, forming the landmark cohort for the primary patency analysis. The overall post‐landmark primary unassisted patency rate for this cohort was 83.6% (95% CI 76.0%–88.9%) at 1 year and 68.2% (95% CI 58.1%–76.3%) at 2 years.

A clear dose–response relationship was observed between the early progression rate and subsequent patency failure (Figure 3). As shown by Kaplan–Meier analysis, patients in the highest tertile of early intima‐media thickness progression had significantly worse patency outcomes compared to those in the lowest tertile (Log‐rank *p* < 0.001). The unadjusted hazard ratio for primary unassisted patency failure was 2.18 (95% CI 0.95–5.01) for the moderate‐progression group and 4.51 (95% CI 2.10–9.68) for the high‐progression group, relative to the low‐progression group.

**FIGURE 3:**
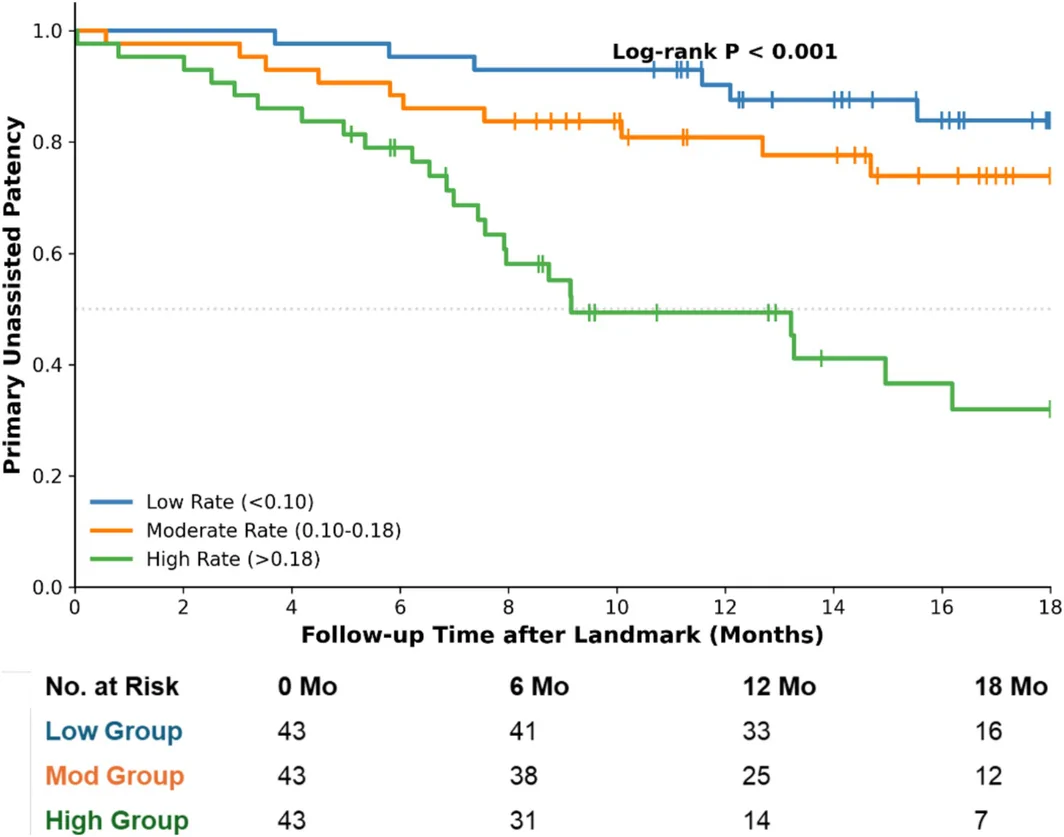
Kaplan–Meier curves for post‐landmark primary unassisted patency, stratified by early intima‐media thickness progression rate tertiles. The analysis includes 129 patients from the 6‐month landmark cohort. Follow‐up time starts from the landmark point (6 months post‐maturation). Patients were stratified based on the intima‐media thickness progression rate calculated from baseline to 6 months. The difference among the groups was statistically significant (Log‐rank *p* < 0.001). Shaded areas represent 95% confidence intervals. The table below the plot shows the number of patients at risk at specified time intervals for each group.

### 3.4 Association of Early Progression With Subsequent Failure

In the univariable Cox regression analysis performed on the landmark cohort, several factors were associated with post‐landmark primary unassisted patency failure (Table 2). Notably, the early intima‐media thickness progression rate (per 0.05 mm/year increment) was the strongest predictor (HR 1.70, 95% CI 1.47–1.96; *p* < 0.001). Other significant predictors included diabetes mellitus (HR 1.75, 95% CI 1.15–2.66; *p* = 0.009) and baseline thickness (per 0.1 mm increment; HR 1.65, 95% CI 1.20–2.27; *p* = 0.002).

**TABLE 2:** Univariable and multivariable Cox proportional hazards analysis for post‐landmark primary unassisted patency failure (*n* = 129).

Variable	Univariable analysis	Multivariable analysis	Adjusted HR (95% CI)	*p*
	HR (95% CI)	*p*		
Demographic and clinical				
Age (per 10‐year increase)	1.15 (0.94–1.41)	0.18	1.09 (0.88–1.35)	0.45
Male sex (vs. female)	1.12 (0.68–1.84)	0.66		
Diabetes Mellitus (Yes vs. No)	1.75 (1.15–2.66)	0.009	1.52 (1.03–2.24)	0.035
Dialysis vintage (per year)	1.08 (0.99–1.18)	0.081	1.05 (0.96–1.15)	0.28
Baseline AV fistula and ultrasound				
AV fistula type (upper arm vs. radiocephalic)	1.21 (0.75–1.95)	0.43		
Initial venous diameter (per mm)	0.85 (0.68–1.07)	0.17	0.91 (0.71–1.16)	0.44
Baseline intima‐media thickness (per 0.1 mm)	1.65 (1.20–2.27)	0.002	1.55 (1.10–2.18)	0.012
Early intima‐media thickness progression rate (per 0.05 mm/year)	1.70 (1.47–1.96)	< 0.001	1.63 (1.41–1.88)	< 0.001

After multivariable adjustment for age, diabetes, dialysis vintage, initial venous diameter, and baseline intima‐media thickness, the early progression rate remained a strong and independent predictor of subsequent patency failure (adjusted HR 1.63 (95% CI 1.41–1.88); *p* < 0.001). In the final model, baseline thickness (aHR 1.55, 95% CI 1.10–2.18; *p* = 0.012) and diabetes mellitus (aHR 1.52, 95% CI 1.03–2.24; *p* = 0.035) also retained independent associations with the outcome. No significant multicollinearity was detected among the variables in the model (all VIFs < 1.8), and the proportional hazards assumption was met (global Schoenfeld test *p* = 0.41).

To explore potential non‐linear relationships, we modeled the early progression rate using restricted cubic splines. The analysis revealed a generally linear and monotonically increasing relationship between the progression rate and the log‐hazard of primary unassisted patency failure, with no evidence of a significant non‐linear effect (*p* for non‐linearity = 0.28), supporting the use of a linear term in the model.

### 3.5 Incremental Prognostic Value of Early Progression

We compared two prognostic models for predicting post‐landmark primary unassisted patency failure. Model 1 included baseline clinical factors (age, diabetes, vintage) and baseline intima‐media thickness. Model 2 added the early progression rate to Model 1.

The addition of the early progression rate significantly improved the model's discriminatory ability. The Harrell's C‐index increased from 0.69 (95% CI 0.62–0.76) for Model 1 to 0.76 (95% CI 0.69–0.83) for Model 2 (P for difference = 0.002). This improvement was consistent across time, as shown by ROC analysis (Figure 4A). At 1‐year post‐landmark, the AUC increased from 0.71 for Model 1 to 0.79 for Model 2.

**FIGURE 4:**
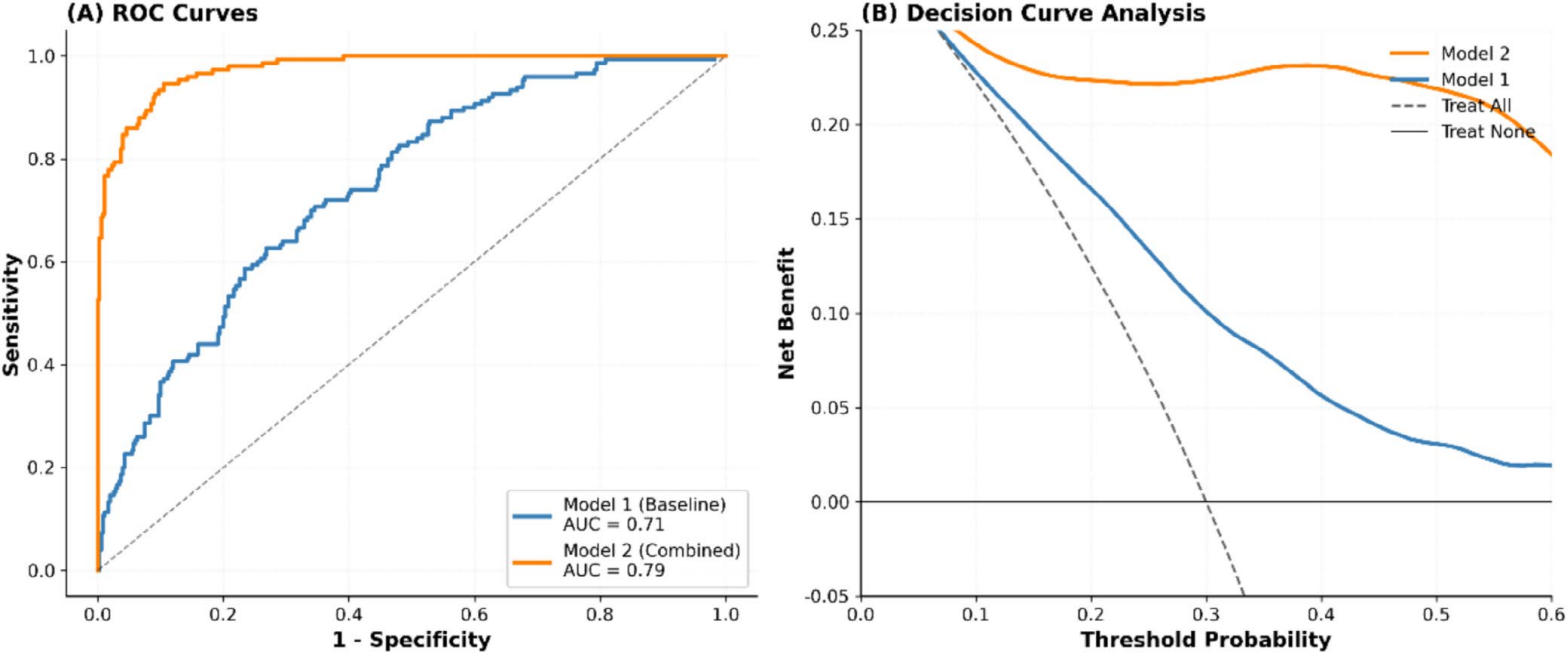
Incremental prognostic value of the early intima‐media thickness progression rate for predicting post‐landmark patency failure. (A) ROC curves for predicting 1‐year post‐landmark primary unassisted patency failure. Model 2 (Baseline + Progression Rate) shows a significantly larger AUC of 0.79 compared to Model 1 (Baseline only, AUC = 0.71), indicating superior discrimination. (B) Decision curve analysis for 1‐year post‐landmark primary unassisted patency failure. Model 2 demonstrates a higher net benefit across a wide range of clinically relevant threshold probabilities (approx. 10%–45%) compared to Model 1, the strategy of treating all patients, or treating no patients, indicating superior clinical utility.

Decision curve analysis demonstrated that Model 2 provided a greater net benefit across a wide range of clinically relevant threshold probabilities (approximately 10% to 45%) compared to Model 1 and the strategies of treating all or no patients (Figure 4B). This indicates that using the early progression rate to inform clinical decisions could lead to better outcomes.

Furthermore, adding the early progression rate led to a significant improvement in risk reclassification. The continuous net reclassification improvement was 0.21 (95% CI 0.05–0.37; *p* = 0.008), and the integrated discrimination improvement was 0.06 (95% CI 0.02–0.10; *p* = 0.004). These metrics suggest that Model 2 more accurately assigns higher risk to patients who experience events and lower risk to those who do not. The calibration plots for both models showed acceptable agreement between predicted and observed probabilities, with Model 2 maintaining good calibration even at higher risk levels.

### 3.6 Sensitivity and Subgroup Analyses

To assess the robustness of our findings, we conducted a secondary landmark analysis at 12 months post‐maturation. Among the 105 patients who were event‐free at 12 months, the progression rate calculated over the first year remained a strong, independent predictor of subsequent patency failure (aHR 1.61, 95% CI 1.34–1.92; *p* < 0.001), consistent with the primary 6‐month analysis.

Subgroup analyses within the 6‐month landmark cohort showed that the prognostic value of the early progression rate was consistent across various predefined subgroups (Figure 5). There was no significant interaction between the progression rate and the presence of diabetes (*p* for interaction = 0.62), fistula type (radiocephalic vs. others; *p* for interaction = 0.78), age, or baseline intima‐media thickness (all *p* for interaction > 0.10).

**FIGURE 5:**
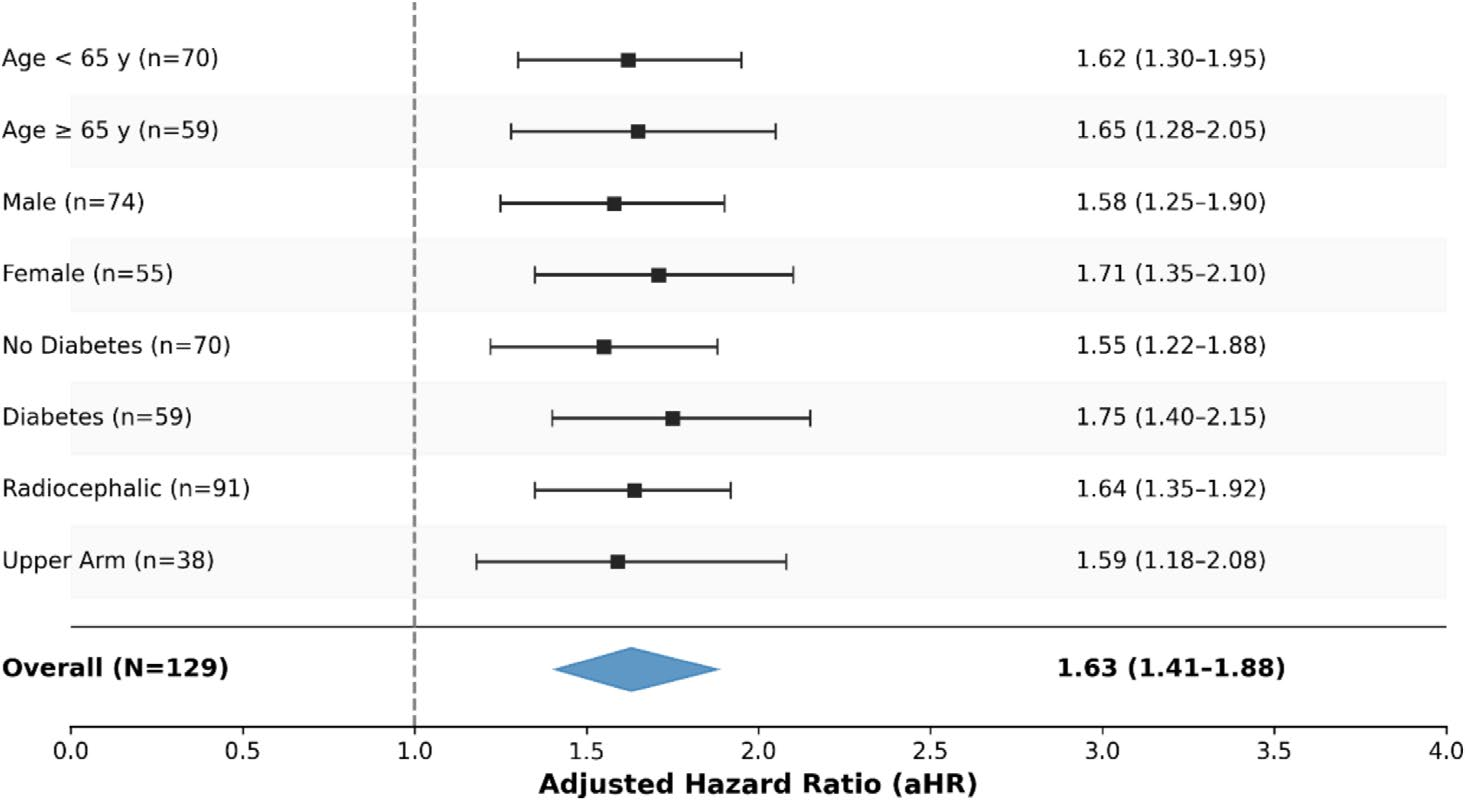
Subgroup analyses of the association between early intima‐media thickness progression rate and post‐landmark patency failure. This forest plot displays the adjusted hazard ratios for the effect of early progression rate within various clinical subgroups. The *p*‐values for interaction were all non‐significant, indicating a consistent effect across all subgroups.

## 4 Discussion

This prospective cohort study provides compelling evidence that shifting the paradigm from static assessment to longitudinal monitoring of venous wall remodeling is critical for predicting AV fistula failure. We found that, in patients who maintained patency for the first 6 months, the early progression rate of intima‐media thickness, measured within the first 6 months post‐maturation, served as a powerful and independent predictor of subsequent primary unassisted patency loss. Specifically, after multivariable adjustment, each 0.05 mm/year increase in the progression rate conferred a 1.63‐fold higher risk of failure. Crucially, the true value of this serial metric lies in its incremental prognostic power. Adding the progression rate to a standard risk model containing established clinical factors and baseline intima‐media thickness led to a significant improvement in risk discrimination (C‐index increased from 0.69 to 0.76), superior risk reclassification (net reclassification improvement = 0.21), and greater clinical utility as demonstrated by decision curve analysis. These findings demonstrate that the trajectory of early venous remodeling, not just its initial state, is a key determinant of long‐term AV fistula viability [11, 12].

The strong predictive power of the intima‐media thickness progression rate is rooted in the fundamental pathophysiology of AV fistula failure. The creation of an access unleashes intense and sustained hemodynamic forces, particularly high flow and disturbed shear stress, on the venous wall, which is structurally unprepared for such an environment [1, 13, 14]. This mechanical stimulus triggers a well‐documented cascade of maladaptive remodeling: endothelial cells become activated and dysfunctional, inflammatory cells are recruited, and underlying vascular smooth muscle cells undergo a critical phenotypic switch from a quiescent, contractile state to a proliferative, synthetic one [15, 16]. These activated smooth muscle cells migrate into the intima and deposit excessive extracellular matrix, forming the neointimal hyperplasia that progressively narrows the lumen [16]. In this context, venous intima‐media thickness acts as a direct, non‐invasive morphological readout of this entire process. More importantly, the rate of progression serves as a proxy for the biological tempo of these events. A faster rate does not simply mean more tissue accumulation; it signifies a more aggressive and accelerated underlying pathology, a more intense inflammatory response, more rapid smooth muscle cell proliferation, and a faster matrix deposition, which naturally leads to the earlier onset of hemodynamically significant stenosis and eventual access failure. Our study provides robust clinical validation for this concept, linking the speed of structural change to clinical outcomes [16, 17].

A particularly insightful finding is that baseline intima‐media thickness and diabetes mellitus remained independent predictors of primary unassisted patency failure even after accounting for the powerful effect of the progression rate. This multi‐layered risk profile suggests that both the initial state of the vessel and the subsequent response to AV fistula creation independently contribute to its fate. A higher baseline thickness likely signifies a pre‐existing structural vulnerability, a vein that is already thickened due to systemic factors or prior subclinical injury, thus having less adaptive reserve. This represents a disadvantageous “starting point.” Concurrently, systemic conditions like diabetes mellitus provide a hostile, pro‐inflammatory, and pro‐fibrotic milieu that “primes” the vein for an exaggerated maladaptive response [4]. Finally, the progression rate quantifies the personalized, real‐time biological “trajectory” of this response. Therefore, our findings paint a clear picture: a patient with a high baseline thickness (a poor start) and a rapid progression rate (a poor trajectory), especially in the context of diabetes, faces a compounded risk of failure. This illustrates the distinct and complementary prognostic value of static and longitudinal risk factors.

The clinical implications of our findings are substantial, offering a potential pathway to refine AV fistula surveillance. Current standard‐of‐care surveillance, which relies on physical examination and reactive monitoring of parameters like access flow, often identifies stenosis only when it is already hemodynamically significant and potentially close to causing thrombosis [18, 19]. Our results advocate for a paradigm shift from this reactive model to a proactive, risk‐stratified strategy. We envision a practical surveillance framework where, following AV fistula maturation, a baseline and a 6‐month intima‐media thickness measurement are integrated into routine care. Patients exhibiting a rapid intima‐media thickness progression rate, for instance, those in the highest tertile of our cohort (> 0.18 mm/year), could be identified as a high‐risk group. This designation would justify triggering them to an intensified surveillance pathway, including more frequent clinical monitoring, lower thresholds for duplex ultrasound evaluation to detect critical stenosis earlier. Such an approach holds the promise of facilitating timely intervention on lesions that progress to hemodynamic significance, thereby preserving access patency and reducing urgent procedures. While mechanistically appealing, the clinical benefit of prophylactic intervention on rapidly progressing yet hemodynamically non‐significant lesions remains controversial and would require dedicated randomized trials to avoid unnecessary procedures. Therefore, our findings primarily support a risk‐adapted surveillance strategy rather than immediate modification of intervention indications.

This study has several strengths, including its prospective design, serial standardized ultrasound measurements, and the use of a landmark analysis to mitigate immortal time bias, which collectively provides a robust estimate of the prognostic value of intima‐media thickness progression. The consistency of our findings across key subgroups and sensitivity analyses further supports the internal validity of our results. Nonetheless, certain limitations should be acknowledged. As a single‐center study, our findings require external validation in larger, multi‐center cohorts. The median follow‐up of 13.2 months may not fully capture very late access failures, and the operator‐dependent nature of ultrasound necessitates standardized protocols for broader application. Furthermore, the potential for residual confounding exists. For instance, future studies should explore the impact of specific systemic factors, such as antiphospholipid antibody status, on venous wall remodeling patterns, as suggested by emerging evidence. Finally, while our data suggest that a risk‐adapted strategy based on progression rate is promising, prospective studies are needed to establish optimal thresholds, and ultimately, randomized controlled trials are required to confirm that such a strategy improves patient‐centered outcomes, such as access longevity, and is cost‐effective.

## 5 Conclusion

In conclusion, this study indicates that the early rate of venous intima‐media thickness progression is a potent and independent predictor of subsequent AV fistula failure in established access. This serial ultrasound parameter provides significant incremental prognostic value over conventional static risk factors, enabling a more accurate identification of high‐risk patients. While serial intima‐media thickness assessment holds considerable promise for guiding risk‐stratified surveillance and optimizing the timing of interventions, its widespread clinical adoption awaits confirmation from multi‐center validation studies and prospective trials of progression‐guided strategies.

## Funding

The authors have nothing to report.

## Ethics Statement

This prospective, single‐center, observational cohort study was approved by the Institutional Review Board of West China TianFu Hospital of Sichuan University (Ethics ID: 2021(961)). All participants provided written informed consent. The study was conducted in accordance with the principles of the Declaration of Helsinki.

## Conflicts of Interest

The authors declare no conflicts of interest.

## Data Availability

The data that support the findings of this study are available on request from the corresponding author. The data are not publicly available due to privacy or ethical restrictions.
